# Anti-inflammatory Effects of Herbal Preparations STW5 and STW5-II in Cytokine-Challenged Normal Human Colon Cells

**DOI:** 10.3389/fphar.2016.00393

**Published:** 2016-10-26

**Authors:** Mathias Schneider, Thomas Efferth, Heba Abdel-Aziz

**Affiliations:** ^1^Department of Pharmaceutical Biology, Johannes Gutenberg University, MainzGermany; ^2^Medical and Clinical Affairs Phytomedicines, Steigerwald Arzneimittelwerk GmbH, Bayer Consumer Health, DarmstadtGermany

**Keywords:** Inflammatory bowel disease, Phytotherapy, Proteomics

## Abstract

Inflammatory bowel diseases (IBD) are chronic relapsing intestinal disorders characterized by up-regulation of pro-inflammatory cytokines followed by invasion of immune cells to the intestinal lamina propria. Standard therapies consist of anti-inflammatory or immunosuppressive drugs. Since clinical efficiency is not satisfactory and the established drugs have massive side effects, new strategies to treat IBD are required. Herein, we investigate the protective effect of the fixed combination herbal preparations STW5 and STW5-II and the contribution of the corresponding single components in an *in vitro* inflammation model. The normal human colon epithelial cell line, NCM460, was treated with STW5, STW5-II or their single components for 4 h followed by experimental conditions comparable to induction of colitis. A pro-inflammatory cytokine cocktail consisting of TNF-α, IL-β, and IFN-γ was used to simulate inflammatory stimuli normally caused by immune cells. The effects on NCM460 cells were investigated by enzyme-linked immunoassay and Proteome Profiler^®^. Levels of IP-10, MCP-1, I-TAC, Groα, and IL-8 were elevated in chemokine-treated cells compared to untreated cells, but significantly reduced upon pretreatment with STW5 or STW5-II. However, the single compounds revealed only little effects on protein expression. Furthermore, we investigated the effect of both combination preparations on pro-inflammatory transcription factors of the STAT family using Western blot. In addition, we tested the effects on upstream MAPK p38. Both, STW5 and STW5-II did not show any effect on MAPK p38, but were effective in reducing phosphorylated levels of STAT1. In conclusion, both combination preparations act in an anti-inflammatory manner by influencing cytokine secretion via reduced activity of the JAK/STAT1 pathway. Relevant differences between STW5 and STW5-II were not found indicating similar efficacies.

## Introduction

Crohn’s disease and UC are the most common forms of IBD with high incidence and prevalence in western civilizations (Karlinger et al., 2000). Both are characterized by a dysregulated balance between pro- and anti-inflammatory processes in the intestinal tract leading to chronic inflammation (Vecchi Brumatti et al., 2014). Although the etiology of IBD remains unclear, genetic, and environmental factors have been implicated in disease pathogenesis (Ferguson et al., 2007; Xavier and Podolsky, 2007).

Medicinal standard therapies comprise anti-inflammatory agents such as 5-aminosalicylate, glucocorticoids, immuno-suppressant drugs, or therapeutic antibodies (Abraham and Cho, 2009; Danese and Fiocchi, 2011). Diagnosis of IBD is based on symptoms and endoscopic evaluation (Kugathasan et al., 2003). The intestinal inflammation is often monitored by clinical indices and invasive techniques (Crama-Bohbouth et al., 1989; Langhorst et al., 2008). Cytokines may act as surrogate markers, since elevated levels have been found in the intestinal mucosa of patients with IBD.

Cytokines are biologically active compounds involved in immune reactions and can be classified as being pro- or anti-inflammatory. They are produced by both resident and migrating cells. A subset of cytokines, called chemokines, is responsible for the migration of immune cells to the site of inflammation (Calixto et al., 2004). Targeting cytokine pathways is a promising target for treating IBD and is already established in the case of anti-tumor necrosis factor-alpha (TNF-α) antibodies. Side effects associated with these therapies, e.g., high risk of infections (Khanna and Feagan, 2015) underscore the need of novel improved treatment strategies.

Some natural products showed promising activities in the treatment of intestinal inflammation (Schneider et al., 2014), and could present an additional treatment option for IBD. STW5 (Iberogast^®^) consists of a fixed combination of nine hydro-alcoholic extracts of medicinal plants which have been traditionally used in Europe to treat diverse gastrointestinal illnesses. It is a well-tolerated herbal medicinal product, clinically used for the treatment of functional dyspepsia and irritable bowel syndrome (Holtmann et al., 2004; Allescher and Wagner, 2007) and shown to be effective in gastroesophageal reflux disease models (Abdel-Aziz et al., 2010, 2015; Patrick, 2011). In animal models of UC, STW5 was able to reduce inflammatory markers and histological changes (Michael et al., 2012; Wadie et al., 2012).

The aim of the present study was to investigate the mechanisms of action of STW5 (fixed combination of nine herbal extracts; Iberogast^®^), the investigational preparation STW5-II (fixed combination of six herbal extracts, lacking three extracts of STW5), and their corresponding single extracts in terms of cytokine secretion from inflamed intestinal cells.

## Materials and Methods

### Substances

STW5 was provided in the form of lyophylisates by Steigerwald Arzneimittelwerk GmbH (Darmstadt, Germany). The preparation consists of the 50% (v/v) hydroethanolic fresh plant extract of *Iberis amara L.* (*Brassicaceae*) whole plants and the 30% hydroethanolic extracts of dried *Melissa officinalis L.* (*Lamiaceae*) leaves, *Matricaria chamomilla* (*Compositae*) flowers, *Carum carvi L.* (*Apiaceae*) fruits, *Mentha piperita L.* (*Lamiaceae*), *Angelica archangelica L.* (*Apiaceae*) roots, *Silybum marianum (L.) Gaertn.* (*Compositae*) fruits, *Chelidonium majus L.* (*Papaveraceae*) herbs, and *Glycyrrhiza glabra L.* (*Leguminosae*) roots. STW5-II (investigational mixture) and the single extracts were also provided as lyophylisates (**Table 1**). The preparations and their single extracts are well characterized according to validated analytical methods. They belong according to the guidelines of the European Medicines Agency to “other herbal substances.” The extraction processes as well as the quality controls were previously described in detail (Kroll and Cordes, 2006; Allam et al., 2015). Briefly, the extracts were produced and their quality controlled according to Good Manufacturing Practice and Good Agricultural Practice of Medicinal and Aromatic Plants (cited and outlined in Kroll and Cordes, 2006). The quality of each extract is tested according to individual specifications, among which is the identity of the used drug (chromatographic fingerprint) and the content of marker substances (Allam et al., 2015) within a defined range, as measured by high performance liquid chromatography or gas-liquid chromatography.

**Table 1:** Overview of used plant extracts: Composition of STW5 and STW5-II.

Code (batch number)	Plant origin	Drug-Extract ratio	Concentrations (μl/ml)	Content in STW5	Content in STW5-II
STW5 (430392)	mixture	–	various	proprietary	–
STW5-II (141008)	Investigational mixture	–	various	proprietary	–
STW6 (13187)	*Iberis amara L.*	1:1.5–2.5	1,5	15%	15%
STW5 KII (13182)	*Mentha piperita L*	1:2.5–3.5	1	5%	10%
STW5 KIII (13176)	*Matricaria chamomilla L.*	1:2–4	3	20%	30%
STW5 KIV (13192)	*Glycyrrhiza glabra L.*	1:2.5–3.5	1	10%	10%
STW5 KV (13175)	*Angelica archangelica L.*	1:2.5–3.5	1	10%	–
STW5 KVI (13188)	*Carum carvi L.*	1:2.5–3.5	2	10%	20%
STW5 KVII (23033)	*Silybum marianum (L.) Gaertn.*	1:2.5–3.5	1	10%	–
STW5 KVIII (13195)	*Melissa officinalis L.*	1:2.5–3.5	1,5	10%	15%
STW5 KIX (23044)	*Chelidonium majus L.*	1:2.5–3.5	1	10%	–

*The used concentrations of the single components depend on the concentrations within STW5-II. In case of KV, KVII, and KIX concentrations of STW5 were used.*

Lyophilisates were freshly re-suspended in Dulbecco’s modified eagle medium (DMEM; Life Technology, Darmstadt, Germany) supplemented with 1% non-essential amino acids (NEAA, Life Technology, Darmstadt, Germany) shortly before use.

### Cell Culture

NCM460 cells were purchased from INCELL Corporation (San Antonio, TX, USA) and cultured in M3 media (San Antonio, TX, USA) supplemented with 10% FBS (fetal bovine serum), 100 U/ml penicillin and 100 μg/ml streptomycin at 37°C in a 95% humidified atmosphere with 5% CO_2_. Cells were passaged at pre-confluent densities using 0.05% trypsin and 0.5 mm EDTA (Invitrogen, Karlsbad, Germany).

### Cell Viability Assay

The cytotoxic effects of the test extracts against NCM460 were assessed after 24 h using a resazurin reduction assay (RRA) according to O’Brien et al. (2000). Cells were seeded in 96-well plates at a density of 10,000 cells per well in 0.1 ml medium. After 24 h cultivation, cells were treated with different concentrations of the plant extracts or DMEM (negative control). Following 24 h incubation at 37°C and 5% CO_2_, 20 μl resazurin working solution (0.01% w/v) were added per well. After another 4 h incubation, the resazurin solution was analyzed by fluorometry using the Infinite M2000 Pro^TM^ plate reader (excitation at 544 nm and emission at 590 nm, Tecan, Crailsheim, Germany). The cytotoxicity of the test extracts was evaluated by comparing values of emission at 590 nm for the treated probes to normalized medium controls. The mean absorbance for the negative control (Medium) was normalized as 100%.

### Proteome Profiler^TM^ Human Cytokine Antibody Array

To evaluate anti-inflammatory effects of plant extracts on NCM460 cells, the cytokine and chemokine production was investigated using the ‘Human Cytokine Array Panel A’ array system obtained from R&D Systems GmbH (Wiesbaden-Nordenstadt, Germany). Following 3-days cultivation of NCM460 cells in a 12 well plate under standard conditions, medium was removed and cells were washed with PBS. Cells were pre-treated with plant extracts for 4 h and subsequently inflammation was induced for 24 h using a human CM consisting of 10 ng/ml TNF-α, 5 ng/ml IL-1β, and 10 ng/ml IFN-γ. After centrifugation, the supernatant was collected and analyzed according to the manufacturer’s instructions.

### Enzyme-Linked Immunoassay

As the “Proteome Profiler^TM^” delivers only semi-quantitative data, enzyme-linked immunosorbent assays were performed using DuoSet Kits from R&D Systems according to the manufacturer’s instructions. Aliquots of 100 μl were pipetted onto the plates coated with the capture antibody. Plates were analyzed using the Infinite M2000 Pro^TM^ plate reader (Tecan) at a wavelength of 450 nm.

### HEK-Blue Reporter Cell Line

HEK293 cell lines stably expressing HEK-Blue-Null1 vector for secreted alkaline phosphatase on a NF-κB promoter were purchased from InvivoGen (San Diego, CA, USA). The cells were cultured according to the manufacturer’s recommendations. The cells were treated with varying concentrations of STW5 or STW5-II for 4 h and then inflammation was induced with TNF-α (10 ng/ml) for 24 h. NF-κB activation was spectrophotometrically detected by measuring secreted embryonic alkaline phosphatase at 630 nm upon Quanti Blue addition (InvivoGen, San Diego, CA, USA). A known NF-κB inhibitor, MG-132 was used as positive control (Zanotto-Filho et al., 2011).

### Cell Extracts

NCM460 cells were seeded at 20,000 cells/cm^2^ in a 6-well plate and grown for 3 days in RPMI media supplemented with 10% FBS, 100 U/ml penicillin, and 100 μg/ml streptomycin at 37°C in a 95% humidified atmosphere with 5% CO_2_. Medium was removed and replaced with DMEM supplemented with 1% NEAA containing or not containing the STW extracts. After 4 h incubation, the medium was removed and cells were induced for various times with human CM consisting of 10 ng/ml TNF-α, 5 ng/ml IL-1β, and 10 ng/ml IFN-γ. Cells were washed with PBS, incubated with cell lysis buffer (New England Biolabs) for 5 min and collected. Lysated cells were sonicated four times for 5 min and centrifuged at 14,000 × *g* for 10 min. The supernatants were used for further analysis.

### TransAM ELISA for NF-κB Activity

NF-κB activity was determined using a commercial ELISA kit (TransAM^®^ NF-κB p65, active motif, La Hulpe, Belgium) according to the manufacturer’s instructions. In brief, cell extracts were added to a 96-well plate in which multiple copies of double stranded DNA were immobilized. After the binding to DNA, NF-κB was detected with HRP-linked antibody. Plates were analyzed using the Infinite M2000 Pro^TM^ plate reader (Tecan) at a wavelength of 450 nm.

### Western Blot

Western Blot was performed as described before (Esquivel-Solís et al., 2009) Protein aliquots (60 μg/well) were loaded on a discontinuous SDS-PAGE for separation. After blotting proteins to a PVDF-membrane, the membrane was incubated overnight with primary antibody; anti-pSTAT1, anti-actin (New England Biolabs). After washing with TBST, membranes were incubated for 1 h with secondary HRP-linked antibody. Before the proteins were detected with Luminata^TM^ Classico Western HRP substrate, the membrane was washed again three times for 5 min with TBST. Documentation of the membranes was performed with ImageJ2 v 2.0 Snap 1.49d software (NIH, Bethesda, MD, USA).

### AP-1 Reporter Assay

NCM460 cells were transfected with signal AP-1 reporter kit (Qiagen) according to manufacturer’s instructions. In brief, cells were suspended and added to a mixture of reporter and transfection reagent. After 24 h of transfection, medium was changed to assay medium (Opti-MEM + 0.5% FBS + 0.1mM NEAA + 1 mM Sodium pyruvate + 100 U/ml penicillin + 100 μg/ml streptomycin) and cells were treated with 10 ng/ml of PMA or CM in mentioned concentrations. PMA is a known inducer of AP-1 activity and was used as a positive control. The AP-1 reporter contains a mixture of inducible AP-1-responsive firefly luciferase construct and constitutively expressing renilla luciferase construct (40:1). The assay was developed by using Dual-Luciferase Reporter Assay System from Promega. Cells were lysed within the wells and luciferase assay reagent was added. Luminescence was measured on an Infinite M2000 Pro^TM^ plate reader (Tecan). After measuring firefly luciferase activity, the stop solution was added and the measurement was repeated to measure renilla luciferase activity. Relative Luciferase activity was determined as luminescence signal of firefly luciferase activity normalized to the signal of renilla luciferase activity.

### MAPK p38 Activity Assay

MAPK p38 activity assay was performed as described before (Huang et al., 1999). NCM460 cells were seeded at 20.000 cells/cm^2^ in 6-well plates and grown for 3 days before treatment. For kinetic measurements, cells were treated with the CM for different periods. For evaluation of STW5 and STW5-II effects, cells were preincubated with the combination preparations for 4 h before adding the CM. Lysed cells were incubated overnight with immobilized anti-pp38 antibody beads. Kinase assays were performed by incubating the immunoprecipitates with ATF-2 fusion protein and ATP for 30 min at 30°C. The pATF-2 content was determined by western blot.

## Results

### Cytotoxicity of STW5 and STW5-II

**FIGURE 1:**
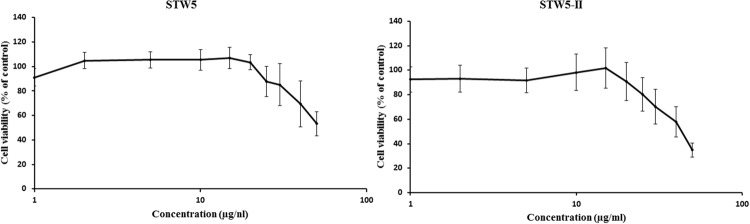
**Cytotoxic effects of STW5 and STW5-II: NCM460 cells were treated with STW5 or STW5-II for 24 h.** Cytotoxicity was measured by resazurin assays by normalizing to untreated control.

Both STW5 and STW5-II did not show cytotoxic effects in a concentration of up to 20 μl/ml (**Figure 1**). For further experiments, only non-cytotoxic concentrations were used.

### STW5 Inhibited Cytokine Release in a Dose-Dependent Manner

**FIGURE 2:**
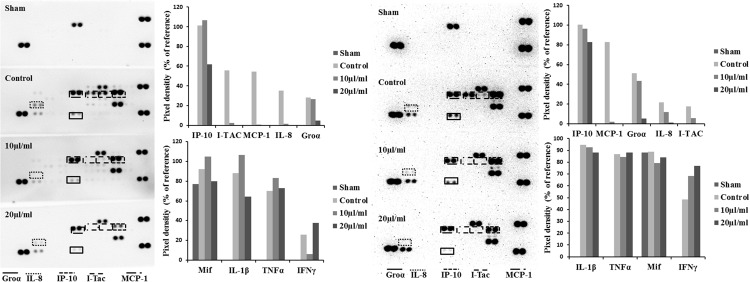
**Effect of STW5 and STW5 on the cytokine profile of NCM460 cells.** Cells were treated with STW5 (left) or STW5-II (right) (10 or 20 μl/ml) for 4 h prior to induction with the CM (10 ng/ml TNF-α, 5 ng/ml IL-1β, and 10 ng/ml IFN-γ) for 24 h. Supernatants were collected and analyzed by Proteome Profiler^®^ and evaluated for Pixel density using ImageJ software.

**FIGURE 3:**
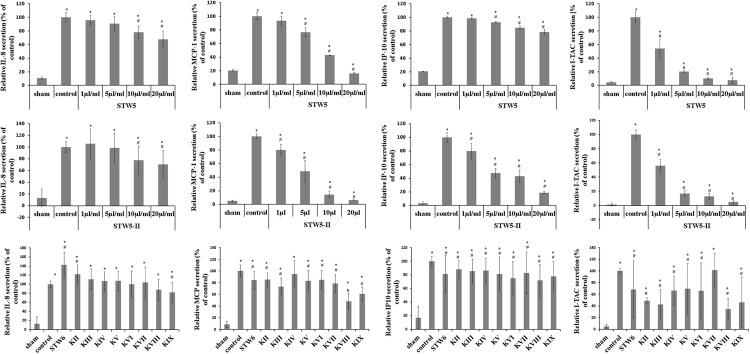
**Dose-dependent effects of STW5, STW5-II, and single plant extracts on cytokine release.** NCM460 cells were treated with STW5 or STW5-II (1; 5; 10; 20 μl/ml) or single plant extract (percentage of STW5-II calculated for 10 μl/ml according to content in herbal preparation) for 4 h prior to induction with the CM (10 ng/ml TNF-α, 5 ng/ml IL-1β, and 10 ng/ml IFN-γ) for 24 h. Supernatants were collected and analyzed by ELISA. # denotes statistical significance at *p* < 0.05 vs. control, * denotes statistical significance at *p* < 0.05 vs. sham.

Cytokine-challenged NCM460 cells showed elevated levels of IL-8, IP-10, MCP-1, and I-TAC. Treatment with both STW5 and STW5-II led to significantly reduced cytokine levels compared to inflamed control as shown by Proteome Profiler. IP-10 showed the highest induction after cytokine treatment. IL-8, MCP-1, and I-TAC were most effectively reduced by STW5 and STW5-II. These four chemokines were chosen for quantitative examination. ELISA analyses revealed dose-dependent effects of STW5 and STW5-II. Inhibition of I-TAC and MCP-1 secretion was most effective and could be reduced at high doses to the level of untreated cells. Due to the quantitative nature of the ELISA, STW5-II showed a dose-dependent effect over 1–20 μl/ml, which was not seen in the Proteome Profiler (**Figure 2**). The single plant extracts showed much weaker effects than the two combination preparations (**Figure 3**).

### Effect on NF-κB Activation

**FIGURE 4:**
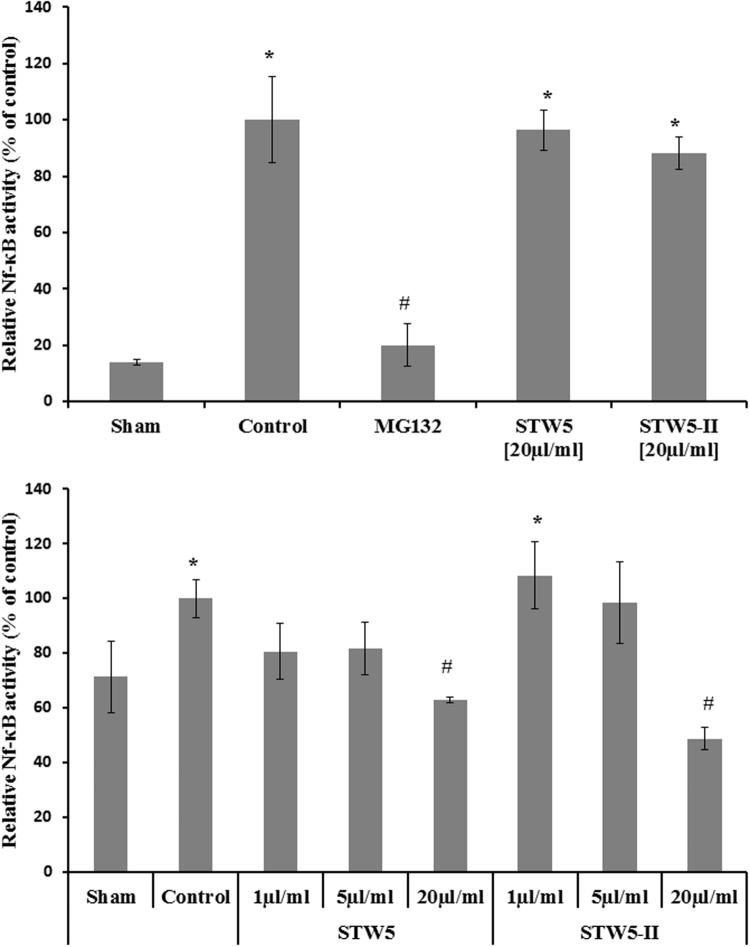
**Effects of STW5 and STW5-II on NF-κB activity: The upper panel displays the NF-κB activity measured by Quanti Blue assay in HEK reporter cells.** The lower panel shows the level of activated NF-κB in NCM460 cell extracts. # denotes statistical significance at *p* < 0.05 vs. control, * denotes statistical significance at *p* < 0.05 vs. sham.

Cytokine-stimulated NCM460 and HEK Blue Null1 reporter cells showed increased NF-κB activity compared to non-stimulated control cells (sham). The levels of activated NF-κB were slightly reduced by both extract combinations (**Figure 4**).

### Effect of Cytokine Mixture on AP-1 Activation

**FIGURE 5:**
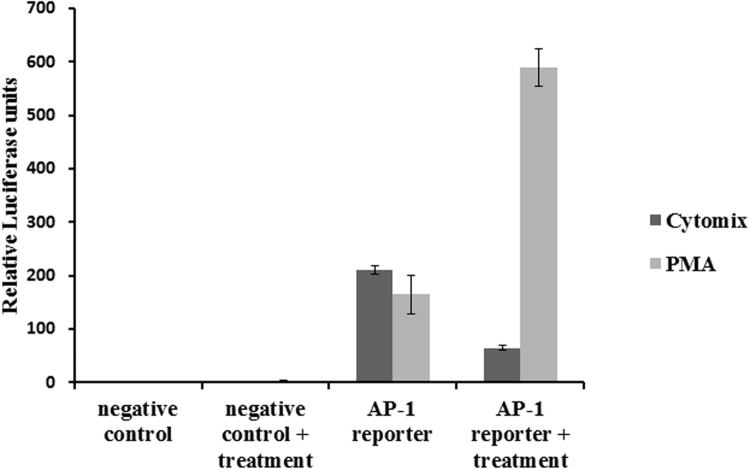
**Effect of the CM on AP-1 activity: NCM460 cells were transfected with luciferase plasmid under the control of AP-1.** Cells were treated with PMA (10 ng/ml) or the CM (10 ng/ml TNF-α, 5 ng/ml IL-1β, and 10 ng/ml IFN-γ) for 24 h. Cell lysates were analyzed for luciferase activity.

Cells transfected with an AP-1 reporter plasmid showed increased luciferase activity. Treatment with inflammatory cytokines reduced the signal (**Figure 5**), rather than increasing it, and therefore did not lead to AP-1 activation. The reporter was also tested with PMA, a standard inducer of AP-1, which led to a marked increase in luciferase activity.

### Effect on MAPK p38

**FIGURE 6:**
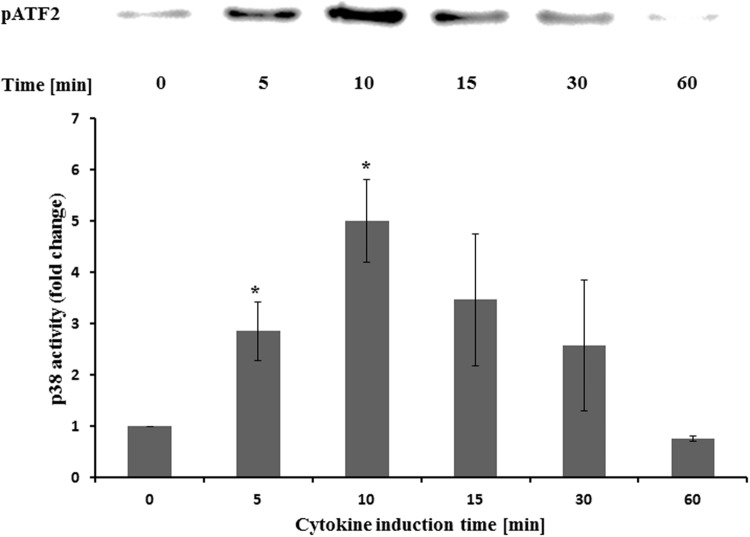
**Effects of STW5 and STW5-II on p38 activity NCM460 cells were treated with the CM (10 ng/ml TNF-α, 5 ng/ml IL-1β, and 10 ng/ml IFN-γ) for the indicated time points prior to harvesting and p38 activity measurement by ATF-2 phosphorylation.** The upper panel shows a representative western blot for pATF-2. The lower panel displays the quantification of three independent western blot experiments (pixel density fold change compared to 0 min; three independent experiments; * denotes statistical significance at *p* < 0.05 vs. 0 min).

**FIGURE 7:**
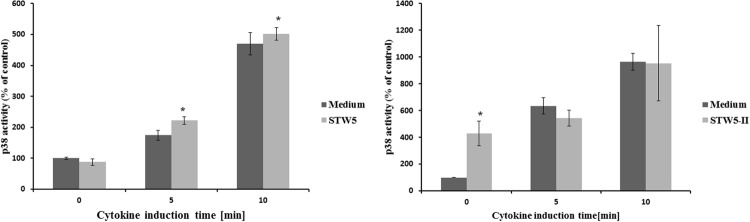
**Effects of single plant extracts on p38 activity: NCM460 cells were treated with STW5-II or STW5 at 20 μl/ml for 1 h prior to induction with the CM (10 ng/ml TNF-α, 5 ng/ml IL-1β, and 10 ng/ml IFN-γ) for the indicated time points.** Cell lysates were used to measure p38 activity by ATF-2 phosphorylation (pixel density fold change compared to 0 min medium treatment; three independent experiments; * denotes statistical significance at *p* < 0.05 vs. medium treatment at the same induction time).

The activity of p38 was determined at different time points after cytokine treatment. It was five times more active after 10 min compared to the untreated control (**Figure 6**). Longer incubation times led to reduced activities. Pretreatment with STW5 or STW5-II did not alter the activity (**Figure 7**).

### Effect on STAT1

**FIGURE 8:**
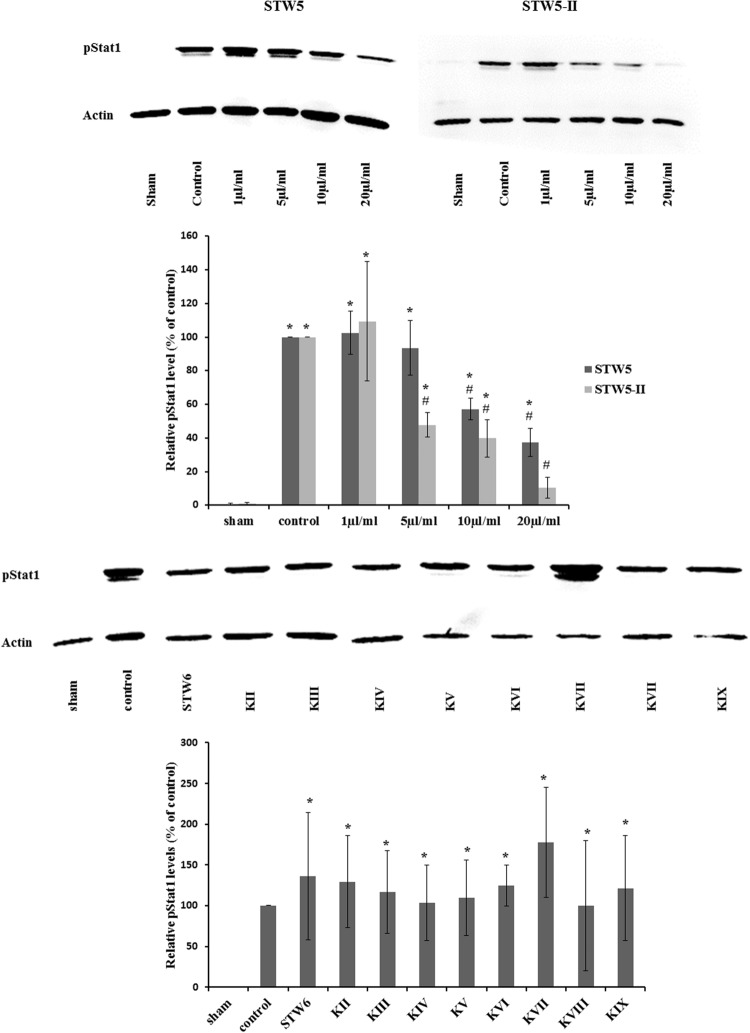
**Effect of STW5, STW5-II, and single plant extracts on Stat1 phosphorylation: NCM460 cells were treated with STW5 or STW5-II at indicated concentrations or single plant extract (percentage of STW5-II calculated for 10 μl/ml according to content in the herbal preparation) for 4 h prior to induction with the CM (10 ng/ml TNF-α, 5 ng/ml IL-1β, and 10 ng/ml IFN-γ) for 30 min.** Cell extracts were analyzed for phosphorylated Stat1 protein by western blotting. The lower panel displays the ratio of pStat1 to actin in relation to the control sample (*n* = three independent experiments; # denotes statistical significance at *p* < 0.05 vs. control, * denotes statistical significance at *p* < 0.05 vs. sham).

Cytokine treatment led to an increased phosphorylation of STAT1 protein compared to non-treated normal controls. Treatment with STW5-II or STW5 reversed this effect in a dose-dependent manner. STW5-II was slightly more effective than STW5 (**Figure 8**). None of the single extracts was able to mimic these effects.

### Statistical Analysis

Results are represented as mean ± SEM. *P*-values were calculated by Student’s *t*-test. Differences were considered as significant at *P* < 0.05.

## Discussion

STW5 (Iberogast^®^) was shown to be effective in a TNBS model of intestinal inflammation via Adenosine A2A receptor activation, and hence inhibition of the TNF-alpha pathway. Furthermore, STW5 increased IL-10 levels in mucosal preparations of mice and reduced TNF-α secretion in LPS-induced human monocytes (Michael et al., 2009, 2012). In a DSS model of inflammation STW5 was able to reduce biochemical markers and attenuated histological parameters. Nevertheless, its influence on the epithelial cells and their contribution to the inflammation process has yet to be investigated.

In the present study, we have evaluated the effects of the clinically established fixed combination preparation STW5 (Iberogast^®^) and the modified STW5-II extract combination, which contains only six of the nine herbal extracts in STW5, for their immunomodulatory effects on human colonic epithelial cells and the underlying molecular mechanisms.

Cell models can be used to mimic an inflammatory state usually by treating the cells with an appropriate stimulus (Romier-Crouzet et al., 2009; Fernandes et al., 2016). To avoid artifacts, we used the normal human colon cell line NCM460 (Moyer et al., 1996) rather than colon cancer cell lines. Therefore, we examined whether NCM460 is responsive to stimuli and useful as a model of inflammation. As inflammatory stimulus, a CM consisting of TNF-α, IL-1β, and IFN-γ was used. These cytokines are known to be released in IBD (Beck and Wallace, 1997; Ciccia et al., 2012) and are effective in inducing inflammatory reactions in cell models (Mueller et al., 2013). First, we profiled the cytokine release of the cells by a membrane-based sandwich immunoassay. NCM460 cells were able to respond and secrete a variety of chemokines, some of which are known to be involved in chemotaxis and were linked to IBD. We decided to focus on IL-8; MCP-1; I-TAC, and IP-10, as those were the ones most strongly modulated in our experimental setting. IL-8 is a powerful neutrophil chemoattractant and activator, whose levels are increased in the mucosa of IBD patients (Mitsuyama et al., 1994). It is also an established inflammatory marker in other cell lines (Ren et al., 2014; Akbari et al., 2015; Noh et al., 2015). Levels of MCP-1 were shown to be increased in IBD (Banks et al., 2003) and were associated with the response toward infliximab therapy (Magnusson et al., 2015). I-TAC facilitates the development of Th17 cells and stimulates Th1 cells to produce IL-6 (Liu et al., 2011). Human intestinal cell lines were shown to produce I-TAC after cytokine stimulation (Marion et al., 2004). IP-10 plays a role in the inflammatory process, as it was shown to be increased in mucosal biopsies from patients with UC or CD (Ostvik et al., 2013) and was induced *in vitro* after IFN-γ stimulation in epithelial cell lines (Dwinell et al., 2001; Marion et al., 2004). It also presents a promising target for treatment of IBD, as a phase II study demonstrated efficacy of anti-IP-10 antibodies in patients suffering from moderate to severe UC (Mayer et al., 2014).

To investigate a dose-effect relationship, we tested the effect of the herbal preparations on chemokine release via ELISA. STW5 was able to reduce IL-8 in a dose-dependent manner, while STW5-II pretreatment led to an increased secretion at low concentrations and decreased release at higher ones. *C. majus L.* (STW5 KIX) was the only single extract that significantly reduced IL-8 levels. MCP-1 release from colon cells was significantly reduced after pretreatment with both STW5 and STW5-II. Furthermore, almost all single extracts except for *G. glabra L.* (STW5 KIV) decreased the secretion of MCP-1 significantly, but not as effectively as STW5 or STW5-II. Both combination preparations reduced the cytokine induced IP-10 secretion. Here STW5-II was a lot more effective than STW5. Interestingly, STW5-II showed dose-dependent effects on the IP-10 release over 1–20 μg/ml, but no great difference between 5 and 10 μg/ml and an immense decrease between 10 and 20 μg/ml. This inconsistency indicates further mechanisms of IP-10 inhibition which occurs only in high doses of STW5-II. All single extracts inhibited IP-10 release comparable to STW5 but not to STW5-II. The secretion of I-TAC was inhibited dose-dependently over 1–20 μg/ml for both extract combinations. Again, chemokine levels were stable over 5–10 μg/ml for STW5-II, supporting the theory of various mechanisms involved in the inhibition.

**FIGURE 9:**
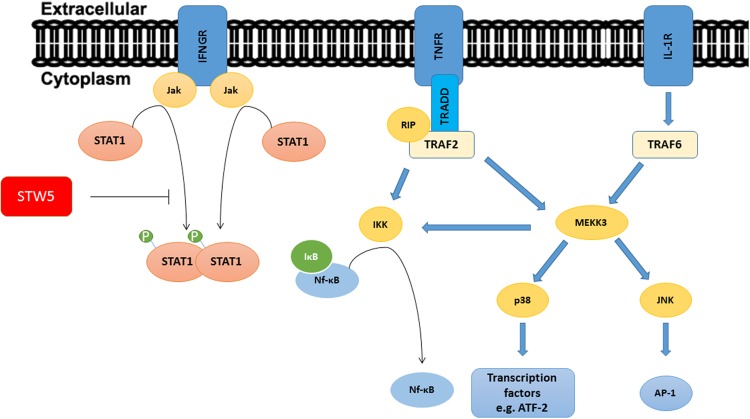
**Signaling pathways of TNF-α, IL-1β, and IFN-γ: The picture shows a simplified representation of the involved pathways.** Recruitment and activation of adaptor proteins lead to the activation of Transcription factors. STW5 and STW5-II reduced the phosphorylation of Stat1.

To examine which signaling pathway might be involved in the anti-inflammatory mechanism of STW5 and STW5-II, we first identified molecules that are affected by the cytokine treatment. Since we used TNF-α, IL-1β, and IFN-γ as stimuli, activated pathways should lead to the activation of transcription factors like AP-1, NF-κB, and STAT proteins. Activation of the IL-1β receptor (IL-1R) or the TNF-α receptor (TNFR) results in recruitment of adaptor proteins which in turn activate secondary proteins. The mitogen-activated protein kinase kinase 3 (MEKK3) is a central kinase in both receptor signaling pathways and is able to activate the MAPKs p38 and c-Jun NH(2)-terminal kinase (JNK; Chen and Goeddel, 2002; Wajant et al., 2003). JNK subsequently activates the transcription factor AP-1 (Weston and Davis, 2002). MEKK3 also plays a pivotal role in activating the IκB kinase [IKK, a kinase that phosphorylates the Inhibitor of kappa B (IκB) and thus causes its degradation and subsequent activation of NF-κB]. Other adaptor proteins in the TNF-α signaling pathway also lead to activation of IKK. Activation of the IFN-γ receptor results in the activation of STAT proteins (Ramana et al., 2015). We decided to focus on these key points in the signaling pathways (**Figure 9**) to gain insights in the molecular mechanisms of STW5. Most of the investigated proteins were shown to be involved in IBD. The MAPK p38 is a key regulator in the activation process of different transcription factors (Chen and Goeddel, 2002; Liacini et al., 2002; Delgado, 2003).

AP-1 regulates cell responses to a variety of stimuli including cytokines (Hess et al., 2004). Because AP-1 was previously shown to be involved in IL-8 release *in vivo* and *in vitro* (Moriyama et al., 2008; Grbic et al., 2012), we examined if AP-1 is also induced with the CM in our experimental setting. The CM was not able to increase the activity of AP-1, indicating that it is not responsible for the cytokine release.

NF-κB is a central transcription factor involved in many cell functions including the regulation of cytokine expression. Cytokines found to be involved in IBD like IL-8 are influenced by NF-κB. Inhibition of the latter was shown to attenuate signs of experimental colitis in mice (Neurath et al., 1996). To determine the activity of NF-κB in NCM460 cells, protein extracts were added to plates in which multiple copies of double stranded consensus DNA were immobilized. Induction of NF-κB was insufficient in this setting, but the results hinted to some activity of STW5 and previous literature data showed a potent effect on NF-κB in colon adenocarcinoma cells (Bonaterra et al., 2013). Therefore, we further examined the influence of cytokines and extract in commercial available HEK-Blue^TM^ Null1 cells which express the SEAP (secreted alkaline phosphatase) reporter gene under the control of the IFN-β minimal promoter fused to five NF-κB and AP-1 binding sites. In these cells, NF-κB activity was sufficiently increased after cytokine treatment. Nevertheless, STW5 and STW5-II had only week effects, not sufficient to explain the inhibition of cytokine release. We concluded that NF-κB activity is not affected by the plant extract combinations in our model.

p38 mitogen-activated protein kinase (MAPK) signaling pathways were shown to be involved in IBD and can be induced by inflammatory cytokines like IL-1β and TNF-α (Feng and Li, 2011). Furthermore, p38 is involved in the activation of transcription factors. To measure the activation of p38, protein extracts were precipitated with immobilized anti-pp38 antibody beads. Activity was measured by the ability of p38 to phosphorylate ATF-2. As expected, the treatment with cytokines increased the activity of p38 which peaked after 10 min induction time. Pretreatment with the extract combinations showed no effect at all during the activation of p38. This indicates that p38 is not affected by STW5 or STW5-II.

STAT proteins are cytoplasmic transcription factors, which dimerize and translocate to the nucleus upon phosphorylation, where they interact with DNA and induce transcription. STAT1 protein expression and phosphorylation were increased in mucosal samples from IBD patients (Schreiber et al., 2002). Also, increased phosphorylation of STAT1 was observed in cell models of IBD (Li et al., 2012; Wang et al., 2012). STAT1 phosphorylation can be induced by IFN-γ, making STAT1 a possible target of the plant extract combinations. We showed that pSTAT1 levels increased after cytokine treatment. Furthermore, pretreatment with STW5 or STW5-II revealed a dose-dependent inhibition over 1–20 μg/ml. STAT3 and STAT5 were also inhibited by the combination preparation. However, their induction was rather week in our experimental setting, therefore their contribution to the overall inflammatory state is probably minor.

Thus, inhibiting STAT1 might explain the reduced cytokine expression, which in turn could partly explain the anti-inflammatory effects of STW5 and STW5-II.

## Conclusion

Human epithelial cells react to inflammatory stimuli (e.g., Th1 cytokines) by releasing a subset of chemokines, which in turn attract immune cells such as macrophages. Our study revealed that the herbal preparation STW5 (Iberogast^®^) and the modified investigational combination STW5-II reversed these effects without affecting NF-κB activity. Further studies showed that the extract combinations reduced STAT1 phosphorylation. We conclude that these herbal preparations may have a therapeutic potential for patients with IBD.

## Author Contributions

MS: Performance of experiments; Writing of Paper. TE: Design of Project; Revision of Paper; Supervision of MS. HA-A: Design of Project; Revision of Paper.

## Conflict of Interest Statement

HA-A is employed by Steigerwald Arzneimittelwerk GmbH. All the other authors declare that the research was conducted in the absence of any commercial or financial relationships that could be construed as a potential conflict of interest.

## Abbreviations

CD: Crohn’s disease
CM: cytokine mixture
ELISA: enzyme-linked immunoassay
IBD: inflammatory bowel disease
IFN: interferon
IL: interleukin
PMA: phorbol 12-myristate 13-acetate
TNF: tumor necrosis factor
UC: ulcerative colitis.
